# How do surgeons decide to refer patients for adjuvant cancer treatment? Protocol for a qualitative study

**DOI:** 10.1186/1748-5908-7-102

**Published:** 2012-10-25

**Authors:** Robin Urquhart, Cynthia Kendell, Joan Sargeant, Gordon Buduhan, Paul Johnson, Daniel Rayson, Eva Grunfeld, Geoffrey A Porter

**Affiliations:** 1 Cancer Outcomes Research Program, Cancer Care Nova Scotia, Halifax, Nova Scotia, Canada; 2 Division of Medical Education, Dalhousie University, Halifax, Nova Scotia, Canada; 3 Continuing Medical Education, Dalhousie University, Halifax, Nova Scotia, Canada; 4 Department of Surgery, Dalhousie University, Halifax, Nova Scotia, Canada; 5 Division of Medical Oncology, Dalhousie University, Halifax, Nova Scotia, Canada; 6 Ontario Institute for Cancer Research, Toronto, Ontario, Canada; 7 Department of Family and Community Medicine, University of Toronto, Toronto, Ontario, Canada; 8 Department of Community Health and Epidemiology, Dalhousie University, Halifax, Nova Scotia, Canada

**Keywords:** Cancer, Surgeon, Referral rates, Qualitative methods, Grounded theory

## Abstract

**Background:**

Non-small cell lung cancer, breast cancer, and colorectal cancer are commonly diagnosed cancers in Canada. Patients diagnosed with early-stage non-small cell lung, breast, or colorectal cancer represent potentially curable populations. For these patients, surgery is the primary mode of treatment, with (neo)adjuvant therapies (*e.g.*, chemotherapy, radiotherapy) recommended according to disease stage. Data from our research in Nova Scotia, as well as others’, demonstrate that a substantial proportion of non-small cell lung cancer and colorectal cancer patients, for whom practice guidelines recommend (neo)adjuvant therapy, are not referred for an oncologist consultation. Conversely, surveillance data and clinical experience suggest that breast cancer patients have much higher referral rates. Since surgery is the primary treatment, the surgeon plays a major role in referring patients to oncologists. Thus, an improved understanding of how surgeons make decisions related to oncology services is important to developing strategies to optimize referral rates. Few studies have examined decision making for (neo)adjuvant therapy from the perspective of the cancer surgeon. This study will use qualitative methods to examine decision-making processes related to referral to oncology services for individuals diagnosed with potentially curable non-small cell lung, breast, or colorectal cancer.

**Methods:**

A qualitative study will be conducted, guided by the principles of grounded theory. The study design is informed by our ongoing research, as well as a model of access to health services. The method of data collection will be in-depth, semi structured interviews. We will attempt to recruit all lung, breast, and/or colorectal cancer surgeons in Nova Scotia (n ≈ 42), with the aim of interviewing a minimum of 34 surgeons. Interviews will be audiotaped and transcribed verbatim. Data will be collected and analyzed concurrently, with two investigators independently coding and analyzing the data. Analysis will involve an inductive, grounded approach using constant comparative analysis.

**Discussion:**

The primary outcomes will be (1) identification of the patient, surgeon, institutional, and health-system factors that influence surgeons’ decisions to refer non-small cell lung, breast, and colorectal cancer patients to oncology services when consideration for (neo)adjuvant therapy is recommended and (2) identification of potential strategies that could optimize referral to oncology for appropriate individuals.

## Background

Lung cancer, breast cancer (BC), and colorectal cancer (CRC) are extremely common in Canada. In 2011, it was estimated these three cancers accounted for 40% of all new cancer diagnoses nationally [1]. These three cancers also represent the leading cancer causes of death in Canada, accounting for 46% of all cancer deaths in 2011 [1]. In Nova Scotia (NS), the incidence rate of BC in females is similar to the national incidence rate, while the incidence rates for lung cancer and CRC are higher in NS for both males and females than national rates [1].

Cancer is a complex disease, often involving multiple healthcare providers from different clinical specialties. For patients with potentially curable disease, comprehensive care involves a sequence of events along a care trajectory that starts at a positive screen or first presentation of signs/symptoms to completion of all adjuvant therapies and ongoing surveillance. In regards to treatment, several distinct modalities may be used singly or in combination in an effort to achieve optimal patient outcomes (*e.g.*, long-term survival). Patients diagnosed with early-stage non-small cell lung cancer (NSCLC), BC, or CRC represent potentially curable populations. For these individuals, surgical resection is the primary mode of potentially curative treatment, with (neo)adjuvant therapies provided according to clinical and/or pathological stage of disease.

Following surgical resection of the cancer, *adjuvant* therapies (*e.g.*, chemotherapy, radiotherapy, hormonal therapy) may be recommended, depending on stage and evidence of benefit, to eradicate loco-regional or systemic microscopic residual disease. Without such therapy, the risk of recurrence with surgical resection alone may be high [2-4]. For some cancers, therapies may be recommended before the surgery in an effort to decrease tumor size (“downstage”) and thus facilitate surgical resection, treat micrometastatic disease, and, in some situations, improve important patient-centered outcomes (*e.g.*, morbidity). In such instances, the therapy is termed *neoadjuvant*, indicating it occurs prior to surgery, to distinguish it from *adjuvant* therapies in the postoperative period.

Unlike many malignancies (*e.g.*, prostate cancer [5]), treatments for NSCLC, BC, and CRC are relatively standardized for the majority of patients receiving potentially curative resection. Indeed, large, randomized clinical trials have demonstrated that receipt of adjuvant chemotherapy improves overall survival in NSCLC, BC, and CRC patients with high-risk disease [6-13]. Consequently, clinical practice guidelines (CPGs), which provide syntheses of the best available evidence, recommend specific (neo)adjuvant therapies for patients with stage II/IIIA NSCLC, stage I–III BC, stage II/III rectal cancer, and stage IIB/III colon cancer [14-19].

### Variations in treatment practices for potentially curable patients

Despite clinical trials demonstrating clear efficacy, the impact of beneficial (neo)adjuvant therapies in patients diagnosed with cancer will ultimately depend on their uptake in patient populations outside of those trials [20]. Health-services research has consistently identified a gap between what is identified as “best practice” (as determined by scientific evidence, largely acquired via randomized clinical trials) and what actually happens in clinical care [21-27]. Many factors have been shown to influence awareness of, agreement with, adoption of, and adherence to best practices (*i.e.*, CPGs). These include the needs and expectations of patients, characteristics of patients and providers, nature of the evidence and its mode of delivery, setting or context of care, and organizational and system constraints or enablers [28-34]. Studies in Canada have revealed variations in (neo)adjuvant therapy rates for appropriate patients, with patient (*e.g.*, age [20,22,27,35-39], comorbidities [36], place of residence [35,37,40]) and health-system (*e.g.*, prevailing wait times [40,41], centralized cancer services [42]) factors associated with these variations. The large variations by age, with older-aged individuals less likely to receive recommended care, demonstrate an age-specific bias toward no treatment, which is not necessarily supported by scientific evidence; in CRC, for example, advanced age has not been associated with lack of a survival benefit nor with increases in chemotherapy-induced toxicities [10,43,44].

While CPGs are meant to guide decision making related to treatment options, and non-receipt of (neo)adjuvant therapy may be entirely appropriate given the circumstances of individual patients, one could argue that having a discussion (or “consultation”) regarding the role of (neo)adjuvant therapy with a physician who specializes in (neo)adjuvant therapies represents the ideal management scenario for patients with potentially curable disease and in whom (neo)adjuvant therapy has been shown to be beneficial. Though there is no defined “ideal” benchmark for referral or consultation rates, both referral to and consultation with an oncologist have been identified as measures of quality care for patients with resected (or resectable) disease [45-47]. Canadian studies examining surgeon referral patterns at single tertiary care institutions report relatively low referral rates to oncology for resected NSCLC patients [48,49] and large variations in referral patterns by patient characteristics for resected CRC patients [36].

Despite the existence of CPGs, clinicians from multiple specialties may have varying perspectives on the benefits/risks of cancer treatment, as well as different interpretations of an increasingly complex evidence base. Researchers have found that surgeons and oncologists report conflicting views on CPG recommendations for adjuvant therapies for BC [50] and CRC [51] patients, while preferred approaches for the management of NSCLC patients vary widely across and within medical and surgical specialties [52]. Thus, the extent to which various specialists become involved in patient care may affect patient decisions related to (neo)adjuvant therapies and the quality of care ultimately received. Referral to an oncologist has been identified as one of the key factors associated with receipt of chemotherapy [53-55].

### An assessment of referral and consultation rates in Nova Scotia

In NS, we have comprehensively examined health-service utilization related to (neo)adjuvant therapy, including referral to an oncologist, oncologist consultation, and receipt of (neo)adjuvant therapy, for all patients diagnosed with CRC over a five-year time period [56]. Our findings revealed that a substantial proportion of patients for whom guidelines recommend consideration for (neo)adjuvant therapy, were not referred to the cancer center for a medical or radiation oncologist consultation. Specifically, we found that approximately one-third of resected stage II/III rectal cancer patients were not referred for a radiation oncology consultation (either in the neoadjuvant or adjuvant settings), and more than 20% of resected stage IIB/III colon cancer patients were not referred for a medical oncology consultation (unpublished data). Once referred, however, the vast majority of patients (>97%) received the consultation. Furthermore, our population-based research demonstrated that the most common reason for not receiving adjuvant chemotherapy in CRC patients for whom chemotherapy is recommended was not having a medical oncology consultation (representing 53% of patients who did not receive chemotherapy) [39]. While these findings may suggest an early selection of patients who were deemed not candidates for (neo)adjuvant therapy, they also point toward the need to better understand the patient, surgeon, institutional, and health-system factors that influence referral to oncology services in NS [39].

For NSCLC, Younis and colleagues [20] found that 73% of resected, stage II–III NSCLC patients in NS in 2005 received a referral to medical oncology for discussion of adjuvant chemotherapy. Crude data estimates from the Nova Scotia Cancer Registry suggest that referral rates for BC are higher than those for NSCLC and CRC, with referral rates in six of nine health districts approaching or reaching 100% (unpublished data). These data corroborate clinical experience, which suggests that surgeons in NS are more likely to refer BC patients to an oncologist for a discussion on the role of adjuvant therapy than NSCLC or CRC patients, despite the fact that many NS surgeons perform both BC and CRC surgery and thus appear to be acting differently for BC versus CRC patients. Taken together, these research and clinical observations reinforce the need to better understand the multiple factors that influence surgical decision making related to (neo)adjuvant therapies.

### Surgeon decision making with respect to cancer treatment(s)

Since surgical resection represents the primary treatment for patients with potentially curative NSCLC, BC, and CRC, the surgeon is chiefly responsible for referring patients to medical and/or radiation oncologists for discussions about (neo)adjuvant therapy. The surgeon also plays a role in the decision to offer (neo)adjuvant therapies (along with the oncologist) and to receive (neo)adjuvant therapies (along with the oncologist and patient). The decision to refer or to recommend therapy is not unlike other patient management decisions, which are invariably made in the midst of uncertainty as the surgeon considers factors such as the patient’s risk of disease recurrence, life expectancy, comorbid conditions, and preferences, as well as information about the treatment and its potential benefits/costs. As such, surgeons may find themselves weighing the probability of clinical benefit versus potential or probable “costs” such as treatment toxicities, patient (non)compliance, patient difficulty accessing cancer treatment facilities, patient lodging and transportation expenditures associated with receiving care (which can represent significant out-of-pocket costs in Canada[57,58]), or even the human or financial resources expended (and possibly denied other patients). For example, population-based studies in the United States [59,60] have demonstrated that a BC patient’s distance to a radiotherapy facility is strongly associated with the type of *surgery* she receives (*e.g.*, breast conservation versus mastectomy; radiotherapy is recommended following breast conservation surgery to reduce recurrence rates [61,62]), suggesting that the structure/centralization of cancer services does influence surgeons’ decisions related to cancer treatment(s). Moreover, health-system factors shown to affect patient utilization of cancer services in Canada, such as prevailing wait times [40,41,63], may also influence surgeons’ decisions to refer patients for a medical or radiation oncology consultation [64].

While population-based studies have demonstrated that variations in referral, consultation, and (neo)adjuvant therapy rates are associated with patient, provider, and health-system factors, the multiple factors that influence the *process of decision making* among cancer surgeons are not well understood. In Canada, research has been conducted into surgeon adoption of sentinel lymph node biopsy for BC and the factors influencing adoption. An initial survey of surgeons who treat BC across Canada found that, for the 39% of surgeon respondents who reported that they do not perform this procedure, the most commonly cited reasons were inadequate resources (53%), lack of evidence to support the procedure (24%), and lack of comfort with the technique (22%) [65], suggesting that factors at multiple levels of the system (*e.g.*, the evidence itself, the provider, and the institution) influence the decision to adopt this evidence-based practice. In a follow-up to this research, Wright and colleagues [66] conducted a qualitative study to gain a more in-depth understanding of the multiple factors that influence sentinel lymph node biopsy adoption. They found that the presence of a high-volume local surgical champion, interprofessional collaboration, institutional support, and the existence of internal hospital protocols and provincial-level CPGs all influenced surgeons’ decisions to adopt this surgical procedure. In regards to decision making about (neo)adjuvant therapies, numerous researchers across Canada, all of whom have reported variations in referral rates and receipt of oncology services, have called for more in-depth study of referral and treatment patterns to improve our ability to develop more effective ways of optimizing care for patients with potentially curable disease [27,36,39,67].

### Rationale for proposed study

Worldwide, it has been estimated that one-third of cancer cases could be prevented and another one-third cured if practices consistently complied with CPGs [68]. In Canada, the Canadian Strategy for Cancer Control estimated that cancer outcomes could be improved by 30% with the appropriate application of existing evidence [69]. Receipt of (neo)adjuvant therapies for resected patients at high risk of recurrence is an essential component of this evidence. While a proportion of patients not referred to oncology services may be appropriately deemed poor candidates for (neo)adjuvant therapy, there may be other factors that influence the decision not to refer and thus prevent patients from receiving a medical or radiation oncologist consultation. Since surgeons act as the main “gatekeeper” to the organized cancer system, understanding how they make decisions related to oncology services is important to developing appropriate strategies and interventions to optimize referral rates and utilization of oncology services.

This study will use qualitative methods to examine decision-making processes related to referral to oncology services for individuals diagnosed with potentially curable NSCLC, BC, and CRC. These cancer sites were chosen for the following reasons:

1. High incidence [1]

2. Existence of clear, relatively standardized treatment practices for these diseases in the (neo)adjuvant setting, with evidence that adherence to best practices improves patient outcomes

3. For NSCLC and CRC, it has been demonstrated that a substantial minority of potentially curable patients in NS are not being referred for an oncology consultation [20,39,56]

4. Based on surveillance data and clinical experience, nearly all potentially curable stage I–III BC patients are referred for an oncology consultation in NS, even though most of the same surgeons are performing CRC surgeries, making BC a valuable and informative disease site to compare and contrast surgeon referral practices

Together, these reasons underscore the need for further study of referral decisions/behaviors in NS. Most researchers who study the adoption and implementation of best practices in healthcare emphasize the necessity of developing a good understanding of the influences on current practice in order to design more effective interventions [30,31,70].

### Research objectives

Through qualitative inquiry, surgeons will be asked to reflect on their decision-making processes related to referral to oncology services for potentially curable NSCLC, BC, and CRC patients, and on the specific factors that influence their decision to refer (or not). The primary objectives are to

1. Identify surgeons’ perspectives on the patient, surgeon, institutional, and health-system factors that influence their decision to refer patients to oncology services;

i. Explore whether surgeons use a mental “threshold of benefit” schema for oncology services and, if so, how they weigh the benefits and costs of an oncology consultation/(neo)adjuvant therapy;

2. Identify the perceived barriers and enablers to referral to oncology services;

3. Explore whether the factors that influence decision making differ by disease site, specifically between patients diagnosed with BC versus patients diagnosed with NSCLC or CRC;

4. Identify potential strategies to promote referral to oncology services for patients for whom (neo)adjuvant therapy is recommended.

Related to objective 1, we will attempt to identify whether and how patient (*e.g.*, age, health status/comorbid conditions, preferences for care), surgeon (attitudes/beliefs related to patients, awareness of evidence/CPGs for adjuvant therapies, level of specialization, and cancer surgery volume), institutional (*e.g.*, academic versus community hospital, structures to facilitate collaborative decision making [*e.g.*, access to multidisciplinary tumor boards], internal/hospital protocols), and health-system (*e.g.*, availability of cancer system resources, accessibility of oncologists/cancer centers, availability/dissemination of provincial-level CPGs) factors influence surgeons’ decision to refer patients to oncology services. Through qualitative inquiry, this study will seek to explore how surgeons evaluate the relative importance of these factors, or weigh the perceived benefits and costs, to come to a decision to refer (or not refer) their NSCLC, BC, or CRC patients for a medical or radiation oncology consultation.

### Conceptual framework

This study is informed by our ongoing research in health services and implementation science [39,71-82], as well as an established model of access to health services [64]. Like all care providers, surgeons operate within a complex healthcare delivery system that is situated in a historical, social, economic, and political context. As a result, decisions related to (neo)adjuvant treatment are likely related to patient factors (*e.g.*, functional status/comorbidities, life expectancy, and risk of disease recurrence), surgeon factors (*e.g.*, level of training/specialization, personal beliefs [52,83]), considerations of treatment benefits and risks, and the broader health system in which they operate. The Penchansky and Thomas [64] model of access to health services provides an approach to understanding access to healthcare that focuses on understanding the “fit” between a patient’s needs and the system’s ability to meet those needs. The model posits that “fit” can be measured through five dimensions, which relate to patient, provider, and health-system factors: availability (volume of physician and other healthcare resources), accessibility (geographic relationship between the users and providers of healthcare), accommodation (organization of care), affordability (costs of providing/receiving care), and acceptability (attitudes and characteristics of patients and providers). More recently, MacKillop [84] presented another important dimension of access: awareness of services and indications for their use. For example, a referring physician must be aware of indications for potentially beneficial services and that those services are available to the patient. Table 1 provides further detail on these six dimensions.

**Table 1:** Six dimensions related to access to health services

**Dimension**	**Examples**
Availability	Resources (personnel, equipment, technology), prevailing wait times
Accessibility	Centralized services, “close to home,” transportation difficulty
Accommodation	Coordination and integration of services, “satellite’ cancer clinics,” telemedicine
Affordability	Funding of cancer services, insurance/drug coverage, indirect patient costs (lodging, transportation)
Acceptability	Patient and provider attitudes toward one another, patient characteristics (*e.g.*, age, sex, comorbid conditions, life expectancy), patient preferences, provider characteristics (*e.g.*, sex, years of practice, level of specialization, surgery volume)
Awareness	Patient and provider awareness of evidence for therapy, clinical practice guidelines, structures that support multidisciplinary dialogue/consultation

### Design and methods

A qualitative research design using semi structured interviews will be used in this study. Qualitative data “document the world from the point of view of the people studied” (p. 165) [85], thereby providing insight into how people make sense of their experiences. Such insight cannot be easily provided by other methods [86]. Qualitative research is often used when there is little existing knowledge (or data) regarding the research topic and to help explain and/or interpret the results of quantitative research [87,88].

### Methodology

This qualitative study will be guided by the principles of grounded theory [89], which attempts to move beyond description and generate a general explanation (a “theory”) of a process or action that is shaped by the views of participants who have experienced the process or action. Grounded theory is “grounded” in the sense that findings tend to be inductively derived from the data and the participants themselves.

According to the methods of grounded theory, concepts and categories are identified and developed as the research is being conducted [89]. While the emphasis of this methodology is on the *inductive* nature of theory building, Strauss and Corbin [89] do not object to the use of pre-existing theory *per se*, but rather in the way it might be used to influence the research process (*e.g.*, by leading the researcher down a path of anticipated findings or assumptions). As Gerson [90] has demonstrated, a sophisticated grounded theory approach rejects the simplistic notion that theory building is entirely inductive (or deductive); instead, theory building occurs in an ongoing dialogue between pre-existing theory and new observations/insights generated from empirical research.

### Semi structured interviews

In-depth, semi structured interviews will be conducted with NS thoracic, breast, and colorectal surgeons to gain their perspectives on factors that influence their decisions to refer patients to oncology services. The value of interviewing is that we can discover understanding about people and their actions that cannot be observed: “[w]e cannot observe how people have organized the world and the meanings they attach to what goes on in the world. We have to ask people questions about those things” (p. 341) [87]. The semi structured interviews will be face-to-face or telephone interviews depending upon practical considerations, such as travel distance to interview a limited number of participants.

Open-ended questions and related probes will be drafted based on the research objectives, team members’ clinical experiences, and the Penchanksy and Thomas model [64], with the latter providing further insight into the patient, surgeon, institutional, and health-system factors influencing utilization of health services. Since decisions around not to refer/treat are made in the context of individual patients, the script will also include scenario-like questions to explore, in a more in-depth manner, how surgeons consider (or weigh) various factors (*e.g.*, relative survival benefit, comorbidities). Interview questions will be adapted for thoracic, breast, and colorectal surgeons since NSCLC, BC, and CRC are different diseases that require different treatment approaches, each with varying degrees of relative benefit. Collectively, the questions will seek to understand how surgeons experience the process of deciding to refer patients for oncology consultation and to identify the factors that influence this process. Two pilot interviews will be conducted with surgeons not part of the research team. These will be audiotaped, transcribed verbatim, and discussed amongst the entire research team to ensure that all topics of interest are explored. The interview script will be refined through these pilot interviews. Consistent with grounded theory principles, the interview guide will be employed in a formative manner and adapted during data collection on the basis of previous interview findings to further explore important concepts and emerging categories [87,91].

One investigator [RU], who is experienced in qualitative methods, will carry out all interviews.

At the beginning of each interview, descriptive information related to the surgeon (*e.g.*, years of practice, level of specialization, cancer surgery volume) and institution (*e.g.*, presence of internal/ hospital protocols, access to multidisciplinary tumor boards) will be collected using a standardized data collection form. Regardless of interviewing mode (face-to-face or telephone), the interviews will be open and characterized by a personal approach, meaning that the interviewer will have a prior understanding of the background and work of the participants, ensure that the participants clearly understand the study objectives and interview procedure, and encourage the participants to express their opinions by explaining that all answers are valid/valuable and will be included in the analysis. Our experience is that such an approach results in encounters that are more like conversations than structured interviews with a series of fixed questions [88]. All interviews will be audiotaped to ensure the data are captured and retrievable in true form and transcribed verbatim by a research coordinator with experience in transcription. The audiotapes and transcripts will be supplemented with field notes (or memos), allowing the interviewer to highlight particularly insightful data and to capture personal reflections during data collection. Transcripts will be verified by listening to the audiotapes. Following each interview, the questions and responses will be reviewed to determine whether or not the issues were answered in sufficient depth and, if not, to revise the questions before the next interview [91].

### Study participants

In grounded theory, the point is to gather enough data to fully develop (or saturate) the explanation [89]. Since there are only a limited number of surgeons in NS who perform NSCLC, BC, and/or CRC surgery (n ≈ 42), we will attempt to recruit all of these surgeons for this study. We will include the data from the pilot interviews with all interview data, with pilot participants’ permission. Recruiting all surgeons will provide a sample with differences in career stage (junior, senior), level of training (general surgeon, surgical oncologist), and practice location (community hospital, academic/tertiary care center). Recognizing that not all surgeons may agree to participate, we aim to interview a minimum of 34 surgeons (80% of relevant surgeons). Our prior experiences surveying rectal cancer surgeons [92] and interviewing breast and colorectal surgeons [unpublished data] in NS suggest this participation rate is achievable.

While recruitment in grounded theory is based on theoretical sampling and saturation, we will attempt to recruit all surgeons due to the relatively small number of surgeons in NS who perform these surgeries. Nonetheless, we will discontinue data collection if we reach saturation before all surgeons are asked to participate. When/if we determine we are nearing theoretical saturation, but some categories (or properties of categories) require further examination, we will make strategic decisions about who will provide the most information-rich source of data to meet our analytic needs (*i.e.*, theoretical sampling).

### Recruitment methods

Two investigators [RU, GAP] will identify all potential participants and initially approach each potential participant in person or via email or telephone to briefly introduce the study and ask whether he/she is willing to participate. If the participant responds in the affirmative, a research coordinator will follow up with the participant to discuss in detail the nature and purpose of the study and to arrange for a time to conduct the informed consent discussion and interview. If a potential participant fails to respond to the initial contact within one week, an investigator [GAP or RU] will follow up with him/her via telephone.

### Anticipated ethical issues

The study poses no serious ethical problems. Ethical approval to conduct this study has been obtained from Capital District Health Authority. Written informed consent will be obtained from each participant. This will include permission to audiotape the sessions and to use anonymized quotes. Any publication will not attribute specific comments to identifiable participants. Participants’ geographic locations (*e.g.*, health region) will not be identified; rather, participants will be described as practicing in community or academic settings.

### Analysis

Data will be collected and analyzed concurrently, allowing emergent concepts and categories to be incorporated and explored in subsequent interviews. An inductive, grounded approach, using constant comparative analysis, will be used for qualitative analysis of interview transcripts and field notes [89]. Grounded theory analysis involves coding; constant comparison; and identification, organization, and refinement of categories. Specifically, the analytic process will entail reading and rereading of transcripts, development of a coding scheme reflecting unique ideas and concepts, application of the coding scheme to the interview text, and grouping of coded text into categories relevant to the study objectives.

Identifying codes is a critical component of the data analysis stage. Coding is defined as the process of grouping participants’ responses into categories that bring together similar ideas, concepts, or themes that the researcher has discovered through familiarity with the interviews and text [91]. Code words reflect the essence of the data and lead to ease of recognition as the number of coded words increase. Consistent with constant comparative analysis, open and axial coding of interview transcripts will occur simultaneously. Open coding entails reading the transcribed interviews line by line in their entirety to identify ideas and concepts and then grouping concepts to form categories and subcategories. This process also involves routinely revisiting previous codes for refining purposes.

Axial coding is used to make connections between the categories and subcategories of codes. Repeating ideas are brought together, leading to the reduction and clustering of categories based on content similarity. These categories will be reviewed and refined based on their relationship to one another and their ability to explain the factors that influence decision-making processes related to referral to oncology services for NSCLC, BC, and CRC. This process will involve ongoing review of the emerging analysis to determine whether and how to expand or merge categories, which will help to ensure that they are reflective of the interview data.

The final stage of analysis will be selective coding, or the detailed development of categories, selection of a core theoretical category, and integration of categories. Specifically, Strauss and Corbin [89] describe selective coding as “the process of selecting the central or core category, systematically relating it to other categories, validating those relationships, and filling in categories that need further refinement and development” (p. 116). The core category should have the analytic strength to “pull the other categories together to form an explanatory whole” (p. 146). However, it must be noted that, for selective coding, theoretical saturation should be reached. Therefore, the analytic process may be limited if we have interviewed all relevant, participating surgeons but have not reached saturation. Nonetheless, identification of categories, linking of categories and subcategories, and clustering around a core or central concept will help us develop a theory (or the building blocks of a theory) that deepens understanding and facilitates action in this important clinical area.

Data will be coded and analyzed independently by two investigators [RU, CK]. These investigators will develop an electronic codebook to guide the coding scheme and subsequent categorization of data; this will be achieved through iterative discussion throughout the data analysis process. The codebook will contain code definitions, sample data illustrating application of the code, and decision rules related to each code. Qualitative analysis will be performed manually, with the assistance of qualitative software (NVivo; QSR International, Cambridge, MA, USA) for data management and to enable comparison and synthesis of codes. To improve the reliability of findings, a third investigator [JS] will review the codebook, all analytic decisions, and sample transcripts or sections of transcripts. We will resolve disagreement among investigators through discussion and, when needed, reexamining transcripts and coded data. To ensure consistency and authenticity of the entire qualitative process, preliminary findings will also be discussed with the three members of the research team who are practicing cancer surgeons in NSCLC, BC, and CRC [GAP, PJ, GB]. The final categories will be presented in diagrams to visually represent the conceptual relationship among categories, tabular form to address each objective, and discussed in relation to the local context as well as the broader scientific literature.

### Strategies for increasing rigor

Numerous steps consistent with qualitative research will be taken to ensure the overall rigor of the proposed study. These include field journal/note-taking during interviews; detailed documentation of methodological and analytic decisions (*i.e.*, an audit trail) to clearly illustrate the means of arriving at the codes and findings and to avoid overgeneralization and unsubstantiated conclusions; systematic data coding and analysis; use of direct quotations to ensure the perspectives of the participants are represented as clearly as possible and to provide the reader with a clearer sense of the evidence on which the findings and interpretations are based; member checking by sending interview participants a summary of the main findings extracted from their interview; review of the data coding process, analytic decisions, and resultant themes by three investigators [RU, CK, JS]; and review and in-person discussion of preliminary findings by three investigators who are practicing cancer surgeons [GAP, PJ, GB]. Importantly, triangulation of findings by the research team and high levels of team involvement throughout data analysis and interpretation enhance rigor by minimizing the chance that important thematic ideas go unnoticed and helping to ensure that the organization of data and the resulting conclusions are transparent.

## Discussion

The primary outcomes of this study will be (1) identification of the patient, surgeon, institutional, and health-system factors that influence surgeons’ decisions to refer NSCLC, BC, and CRC patients to oncology services when consideration for (neo)adjuvant therapy is recommended and (2) identification of potential strategies (interventions) that could increase referral to oncology for appropriate individuals. The findings from this study will help fill an important gap in our understanding of how surgeons make decisions in regards to their patients and how (and the extent to which) organizational and health-system factors can influence decision making during the clinical encounter. As such, the knowledge gained will support the development and implementation of appropriate strategies to optimize referral rates and access to oncology services. Depending on the surgeon-identified influential factors, strategies could target the patient level (*e.g.*, patient decision aids, shared decision-making models), surgeon level (*e.g.*, focused educational activities, audit and feedback), and/or organizational/policy level (*e.g.*, performance monitoring systems, expansion of multidisciplinary tumor boards, use of telemedicine) of the health system. For example, expansion of multidisciplinary tumor boards across the province, or introduction of telemedicine services to permit “just-in-time” surgeon and/or patient consultation with an oncologist at a cancer center, may increase collaborative decision making and optimize referrals to oncology services [39], and support co-management options for community-based surgeons who do not have regular opportunities to interact with other cancer specialists [93].

In summary, receipt of (neo)adjuvant therapies for appropriate patients is an essential component of evidence-based practice for NSCLC, BC, and CRC patients. In NS, prior research indicates that a substantial proportion of NSCLC and CRC patients for whom CPGs recommend consideration for (neo)adjuvant therapy are not referred for a medical or radiation oncologist consultation. Since surgeons act as a “gatekeeper” to the organized cancer system, understanding how they make decisions related to oncology services is important to developing appropriate strategies and interventions to optimize referral rates and improve access to these services.

## Competing interests

The authors have no conflicts of interest to declare.

## Authors’ contributions

RU and GAP conceived the idea for this study, led the intellectual development and protocol writing, and will be primarily responsible for its conduct. CK, JS, GB, PJ, DR, and EG all contributed to the drafting and development of the study. All authors read and approved the final manuscript.
